# Supplementary material for: The Protective Role of Microglial PPARα in Diabetic Retinal Neurodegeneration and Neurovascular Dysfunction
Source: Cells. 2022 Dec 1;11(23):3869. doi: 10.3390/cells11233869 (PMC9739170; doi:10.3390/cells11233869)
Supplement: Supplementary file 1 [file cells-11-03869-s001.zip › cells-2006060-supplementary.pdf]

Supplemental Figure S1 Efficiency analysis of PPARα siRNA knocking down in Human microglia cell HMC3.

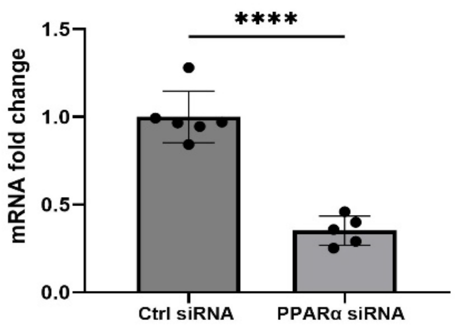

Supplemental Figure S1. siRNA knockdown efficiency analysis. HMC3 cells were transfected with *Pparα* siRNA or scramble siRNA. Forty-eight hours after transfection, cDNA was synthesized following RNA purification. Human *Pparα* was amplified using SYBR Green with the primers: 5'-GTTCAATGCACTGGAACTGG -3' (Forward) and 5'-GACGATCTCCACAGCAAATG-3' (Reverse). β-actin was used as the housekeeping control. Human β-actin forward primer is 5'-GCCGGCTTCGCGGGCGACGA-3'. Human β-actin reverse primer is 5'-GCCACACGCAGCTCATTGTAGA-3'. n=5-6. Student's t-test. ****$p<0.0001$. Data are presented as mean±SD.

Table S1 Antibodies

Name	Vendor	Dilution	Identifier
Rabbit Anti-Iba-1	Wako	1:100-300	Cat#: 01919741,<br>RRID: AB_839504
Rat Anti-CD11b	Cell signaling	1:100	Cat#: 46512
Rabbit Anti-STING	Proteintech	1:100	Cat#: 19851-1-AP<br>RRID: AB_10665370
Rabbit Anti- TNFα	Abcam	1:1000	Cat#: ab9739<br>RRID: AB_308774
Rabbit Anti- TNFα	Abcam	1:50	Cat#: ab1793<br>RRID:AB_302615
Rabbit Anti- PPARα	Novus Biologicals	1:1000	RRID: AB_10001414
HRP conjugated Goat Anti- β actin	Santa Cruz	1:3000	Cat# sc-47778HRP<br>RRID: AB_626632
Alexa Fluor 488-conjugated Donkey Anti-rabbit	Jackson ImmunoResearch Labs	1:500	Cat#: 711-545-152,<br>RRID:AB_2313584
Alexa Fluor 594-conjugated Donkey Anti-rabbit	Jackson ImmunoResearch Labs	1:500	Cat#: 711-585-152,<br>RRID:AB_2340621
Alexa Fluor 594-conjugated Donkey Anti-rat	Jackson ImmunoResearch Labs	1:500	Cat#: 712-585-153,<br>RRID:AB_2340689
Peroxidase conjugated Goat Anti-rabbit	Vector Laboratories	1:2000	Cat#: PI-1000,<br>RRID:AB_2336198
Peroxidase conjugated Horse Anti-mouse	Vector Laboratories	1:2000	Cat#: PI-2000,<br>RRID:AB_2336177
